# Accuracy of Outcome Anticipation, But Not Gaze Behavior, Differs Against Left- and Right-Handed Penalties in Team-Handball Goalkeeping

**DOI:** 10.3389/fpsyg.2015.01820

**Published:** 2015-12-01

**Authors:** Florian Loffing, Florian Sölter, Norbert Hagemann, Bernd Strauss

**Affiliations:** ^1^Department of Psychology and Society, Institute of Sports and Sports Science, University of KasselKassel, Germany; ^2^Department of Sport Psychology, Institute of Sport and Exercise Sciences, University of MuensterMuenster, Germany

**Keywords:** handedness, perceptual asymmetry, eye-tracking, visual search, throwing, perceptual-cognitive expertise

## Abstract

Low perceptual familiarity with relatively rarer left-handed as opposed to more common right-handed individuals may result in athletes' poorer ability to anticipate the former's action intentions. Part of such left-right asymmetry in visual anticipation could be due to an inefficient gaze strategy during confrontation with left-handed individuals. To exemplify, observers may not mirror their gaze when viewing left- vs. right-handed actions but preferentially fixate on an opponent's right body side, irrespective of an opponent's handedness, owing to the predominant exposure to right-handed actions. So far empirical verification of such assumption, however, is lacking. Here we report on an experiment where team-handball goalkeepers' and non-goalkeepers' gaze behavior was recorded while they predicted throw direction of left- and right-handed 7-m penalties shown as videos on a computer monitor. As expected, goalkeepers were considerably more accurate than non-goalkeepers and prediction was better against right- than left-handed penalties. However, there was no indication of differences in gaze measures (i.e., number of fixations, overall and final fixation duration, time-course of horizontal or vertical fixation deviation) as a function of skill group or the penalty-takers' handedness. Findings suggest that inferior anticipation of left-handed compared to right-handed individuals' action intentions may not be associated with misalignment in gaze behavior. Rather, albeit looking similarly, accuracy differences could be due to observers' differential ability of picking up and interpreting the visual information provided by left- vs. right-handed movements.

## Introduction

Left-right perceptual asymmetries are evident in humans, for example, when judging emotions from others' faces (Voyer et al., 2012), bisecting lines (Jewell and McCourt, 2000), viewing natural scenes (Nuthmann and Matthias, 2014), bumping into doorways (Nicholls et al., 2007), or making aesthetic preference judgments (e.g., Chokron and De Agostini, 2000; Maass et al., 2007; Friedrich et al., 2014). While these phenomena may, at least in parts, be explained by innate neurological mechanisms such as right-hemispheric specialization, environmental factors such as the predominance of human right-handedness and associated behavioral conventions are thought to be relevant as well (Marzoli et al., 2014).

The considerable imbalance in the frequency of left- and right-handed individuals may elicit left-right asymmetries in the pick-up and processing of visual information. Leaving aside subtle variations due to task (Loffing et al., 2014), sex (Gilbert and Wysocki, 1992), geography (Perelle and Ehrman, 1994), culture, or religion (Fagard and Dahmen, 2004; Faurie et al., 2005), only about 10–12% of the normal population are estimated to be left-handed (Llaurens et al., 2009). In sports, left-handedness is also rarer than right-handedness (Grouios, 2004; Loffing and Hagemann, 2012). However, compared to the normal population estimate, a higher prevalence of left-handedness has been reported at the elite level of interactive sports such as in cricket for bowling (Edwards and Beaton, 1996) and batting (Brooks et al., 2004), in baseball for pitching and hitting (Goldstein and Young, 1996), table tennis (Raymond et al., 1996), fencing (Azémar et al., 1983) or tennis (Loffing et al., 2012a), suggesting that left-handed athletes might have an advantage in these domains (e.g., see Loffing and Hagemann, 2012, for a review).

Albeit forming a minority, left-handed players are also quite common in team-handball. This is because it is optimal for a team to have at least two left-handed players on the field; specifically on the right wing and right backcourt position (Schorer et al., 2009). From these positions, left-handed players have better shooting angle toward the goal (the same applies to right-handed players for positions on the left side of a field). Coaches strive to occupy at least these positions with players who throw left-handed. Since in most instances the remaining four field-players and the goalkeeper are right-handed, at least 2 out of 7 players can be expected to be left-handed in professional team-handball. Lobinger et al. (2014) recently reported that 133 out of 308 successfully converted penalties (from a total of 419 awarded penalties) at the European Handball Championship 2010 were shot left-handed. Own investigation of handedness distribution among the top goal-scorers at the team-handball World Championships from 2005 to 2015 revealed that 25.64–44.44% of players were left-handed; indicating that left-handedness is a relevant issue for team-handball goalkeeping (see Table 1).

**Table 1:** **Handedness of the top goal-scorers at the team-handball World Championships in 2005–2015**.

**Year**	***N***	**Number (percentage)**	
		**Left-handed**	**Right-handed**
2005	37	11 (29.73%)	26 (70.27%)
2007	39	10 (25.64%)	29 (74.36%)
2009	38	10 (26.32%)	28 (73.68%)
2011	40	14 (35.00%)	26 (65.00%)
2013	40	17 (42.50%)	23 (57.50%)
2015	36	16 (44.44%)	20 (55.56%)

*Lists of top goal-scorers were retrieved from the official website of the International Handball Federation (www.ihf.info). Handedness of players was obtained from searches on the web (e.g., pictures, videos, player biographies)*.

Predominant exposure to right-handed individuals in daily routine and sports may bias visual attention toward others' right body side (i.e., toward the left from an observer's perspective; e.g., Marzoli et al., 2014, 2015) since information relevant for social interaction may be most likely available on this side. Such bias could be further intensified by “rightwards” social conventions such as shaking hands with the right but not the left hand (McManus, 2002). As a consequence, gaze may not be optimally oriented when watching left-handed actions. Specifically, an observer's gaze behavior could be asymmetrical along the vertical axis when faced with (mirrored, but otherwise identical) left-handed as opposed to right-handed actions. Assuming further that gaze should be optimally aligned when viewing more familiar right-handed than left-handed actions, observers may miss out visual information relevant, for instance, for judging left-handed individuals' action intentions during social interactions.

Dynamic sport situations provide an ideal arena for studying the consequences of such left-right asymmetries for performance. The spatiotemporal constraints acting upon athletes necessitate that they are able to anticipate an opponent's intentions prior to a critical event (e.g., the moment of ball release in team-handball penalties, cricket bowling or baseball pitching; Yarrow et al., 2009). Application of gaze strategies allowing an efficient pick-up of predictive visual cues is considered one prerequisite for successful anticipation and such strategies seem particularly characteristic of expert compared to less skilled performers (Mann et al., 2007). In this regard, low perceptual familiarity with stimuli such as left-handed actions may be assumed to result in less efficient gaze behavior and less accurate predictions of action outcomes compared to perceptually more familiar right-handed actions.

Indeed, previous work demonstrated that observers have more difficulty anticipating left- as opposed to right-handed action intentions (Hagemann, 2009; Loffing et al., 2012b, 2015). For example, Hagemann (2009) asked novice, intermediate and expert tennis players (18 left- and 18 right-handed players per group) to visually anticipate the outcome of left- and right-handed tennis strokes occluded at the moment of racket-ball-contact and presented as videos on a computer monitor. To exclude potential differences in original left- and right-handed strokes as an alternative explanation for a handedness effect in anticipation performance (e.g., a limitation in McMorris and Colenso, 1996), half of the trials showed horizontally mirrored versions of strokes (i.e., original left-/right-handed strokes were also presented as inverted right-/left-handed strokes). Analysis revealed that mean prediction error was lower against right- than left-handed opponents in the videos and that this difference was largest in the group of expert players. Furthermore, both left- and right-handed participants had similar difficulty anticipating left-handed strokes, suggesting that an observer's handedness may not play a key role to explain the handedness effect. Rather, the effect seems due to the relative rarity of left- compared to right-handed individuals and resulting inequality in perceptual familiarity with left- and right-handed actions (negative perceptual frequency effect, Hagemann, 2009). Evidence in support of this assumption comes from a perceptual training study with novices in team-handball goalkeeping. Groups who practiced exclusively against left- or right-handed penalty-takers during a three session training intervention demonstrated hand-specific improvements in prediction accuracy from pre- to post-test (Schorer et al., 2012).

Apart from the demonstration of the handedness effect, its underlying perceptual-cognitive mechanisms, however, are only poorly understood. As far as we know there is only one study available in the literature which examined athletes' gaze behavior during the prediction of left- and right-handed action outcomes in volleyball (Neumaier, 1983). Findings from that work suggest that gaze may not be adequately adjusted to an opponent's handedness. Specifically, visual fixations concentrated around an attacker's right arm-shoulder area irrespective of his handedness for hitting volleyball. As an important limitation, however, in contrast to recent research accuracy did not differ between left- vs. right-handed attacks and the content of left- and right-handed stimuli was not kept symmetrical (Hagemann, 2009; Loffing et al., 2012b, 2015; Schorer et al., 2012).

**Figure 1:**
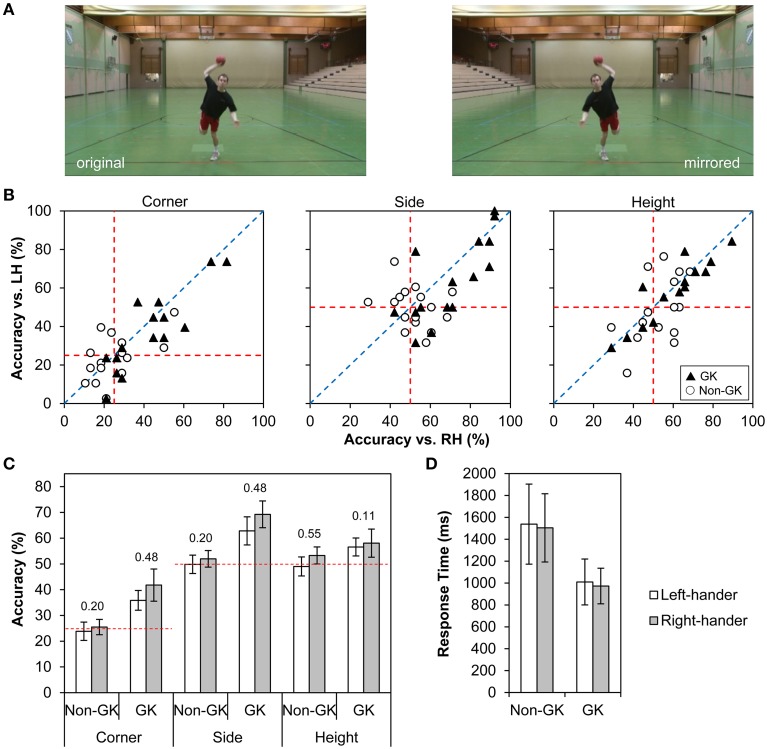
**(A)** Screenshot of the final frame of a video in original and horizontally mirrored orientation. **(B)** Mean prediction accuracy against a right- (RH) vs. left-handed (LH) version of an otherwise identical penalty (i.e., 16 different videos) separately for corner, side and height predictions in goalkeepers (GK; ▴) and non-goalkeepers (Non-GK; ○). Symbols below the diagonal dotted blue line represent penalties where predictions were better against a right- than left-handed version (and *vice versa*). Red dotted lines indicate chance level for right- (vertical lines) and left-handed penalties (horizontal lines). Symbols toward the right (left) and/or above (below) these lines are indicative of above (below) chance performance against right- and left-handed penalties, respectively. **(C)** Mean prediction accuracy for corner, side and height predictions across all left- and right-handed penalties separately for goalkeepers (GK) and non-goalkeepers (Non-GK). Horizontal dashed red lines represent chance levels for corner (25%), side and height (both 50%) predictions. Error bars represent 95% confidence intervals associated with each mean value such that error bars not including the red lines indicate above chance performance. Values above bars are Cohen's standardized effect sizes *d*_*z*_ for differences in accuracy against left- vs. right-handed penalties within goalkeepers and non-goalkeepers. **(D)** Mean response time (±95% confidence intervals) against left- and right-handed penalties separately for goalkeepers and non-goalkeepers.

Here we sought to examine whether hypothesized lower accuracy for the prediction of left- than right-handed action outcomes is associated with corresponding maladjustment in gaze behavior. We chose the 7-m penalty in team-handball as test situation because, among others, the goalkeepers' ability to anticipate a thrower's shot intention has been highlighted as one key feature for successful interception (e.g., Bideau et al., 2004; Cañal-Bruland and Schmidt, 2009; Schorer and Baker, 2009; Bourne et al., 2013; Loffing and Hagemann, 2014). In the experiment, we recorded team-handball goalkeepers' and non-goalkeepers' eye-movements while they watched videos of left- and right-handed 7-m penalties and predicted their directional outcome. To ensure that content of left- and right-handed penalties was symmetrical along the vertical midline of videos, each video was presented in original and horizontally mirrored orientation (e.g., an original right-handed throw toward the top left corner of a goal was also shown as a left-handed throw toward the top right corner; for an illustration see Figure 1A). This methodological step allowed full comparability of left- and right-handed stimuli as a requirement for proper interpretation of possible differences in gaze behavior as a function of an opponent's handedness. We included two differently skilled groups into the protocol to verify that our experiment was capable of capturing components of handball-specific perceptual-cognitive expertise (Mann et al., 2007).

Based on previous findings (Hagemann, 2009; Loffing et al., 2012b, 2015; Schorer et al., 2012) and assuming that participants have considerably lower perceptual familiarity with left- as opposed to right-handed actions (Gilbert and Wysocki, 1992; Loffing et al., 2014), we hypothesized that accuracy would be lower against left- than right-handed penalties. Further, we hypothesized that goalkeepers would be more accurate and respond earlier than non-goalkeepers (Loffing and Hagemann, 2014), but we did not make a specific prediction with regard to an interaction between the factors skill and throwers' handedness given the heterogeneity of previous findings (see Loffing et al., 2015, for a related discussion). Recording of gaze behavior mainly served an exploratory purpose particularly with regard to a possible effect of throwers' handedness. In light of meta-analytic findings (Mann et al., 2007), however, goalkeepers were expected to demonstrate fewer fixations of longer duration as opposed to non-goalkeepers (cf. Savelsbergh et al., 2002, in soccer goalkeeping).

## Materials and methods

### Ethics statement

The study protocol was approved by the ethics committee at the Department of Social Sciences at the University of Kassel. The work was conducted according to the principles specified in the Declaration of Helsinki. Participants signed written informed consent prior to the start of testing. They did not receive any reward for their participation.

### Participants

Nineteen team-handball goalkeepers (age: *M* = 26.47 years, *SD* = 5.99; experience: *M* = 16.06 years, *SD* = 5.35; highest league ever played: 1st–5th in Germany) and 19 non-goalkeepers (age: *M* = 25.32 years, *SD* = 2.51; no competitive experience in team-handball) took voluntarily part in the experiment. All participants were male, naïve as to the purpose of the study and reported to have no or corrected-to-normal vision. To obtain an estimate of participants' handedness, in a post-experiment questionnaire they were asked to indicate the hand they would use for throwing a ball in team-handball as well as to fill out a slightly modified German version (see Loffing et al., 2014, for details) of Oldfield's (1971) Edinburgh Handedness Inventory based on which laterality quotients (LQ) were calculated ranging from −100 (completely left-handed) to +100 (completely right-handed). Accordingly, all goalkeepers reported to be right-handed (LQ: range = +50 to +100, *M* = 89.44, *SD* = 16.37), whereas two non-goalkeepers preferred throwing a ball left-handed (LQ in left-handed individuals: −90 and +57.14; LQ in right-handed individuals: range = 0 to +100, *M* = 86.98, *SD* = 24.5).

### Apparatus and stimuli

Throws of three right- and three left-handed penalty-takers were recorded with a digital video camera (SONY HDR-FX1000e) from a team-handball goalkeepers' perspective. Shots were directed to one of the four corners of a regular handball goal. For the creation of experimental clips, four shots (one to each corner) from two left- and two right-handed players were selected, thus totaling 16 penalties. From then on, video editing steps included temporal occlusion of actions 40 ms prior to ball release and the creation of horizontally mirrored versions of shots. The latter step was necessary to control left- and right-handed penalty-takers' kinematics (e.g., kinematic differences have been reported for left- vs. right-handed pitchers in baseball; Werner et al., 2010) for proper analysis of handedness effects in gaze behavior and visual anticipation (e.g., Hagemann, 2009; Loffing et al., 2015). Video editing resulted in a total of 32 different experimental clips. Videos were 1280 × 720 pixels (width × height) in size with a frame rate of 25 fps and duration between 2920 and 3000 ms.

The experiment was programmed with the software Experiment Builder (*SR Research*) and a stationary desktop mounted eye-tracker (EyeLink 1000, *SR Research*; capture rate: 1000 Hz) was used for binocular tracking of the participants' gaze. Videos were displayed on a 19″-CRT-monitor (ViewSonic® G90f) with a resolution of 1280 × 1024 pixels (width × height) and a refresh rate of 75 Hz. During testing, participants were seated in front of the monitor (eye-to-screen distance: 53 cm) with their head being positioned in a chin-rest.

### Procedure

Participants were provided with standardized written instructions about procedure and task. They then completed four familiarization trials where they were shown two shots of one left- and right-handed penalty-taker each directed to a different corner of a goal. These video models were different to those included in the experimental clips. Each video stopped 40 ms before ball release and then the screen turned black. The participants' task was to predict as accurately as possible throw direction from their perspective as goalkeeper by pressing one of four response keys on a QWERTZ keyboard (E: top left, C: bottom left, O: top right, M: bottom right). There was no time limit for responding; nevertheless we also recorded and analyzed response time. Once a prediction was made, participants received feedback on the actual shot outcome (text info displayed for 2 s).

After the familiarization trials and prior to the start of the experiment, the eye-tracker was adjusted to a participant's eyes in a two-step calibration/validation procedure where participants fixated points that were presented in random order (pacing interval = 1000 ms) on a 3 × 3 grid. Mean absolute average errors from validation of the left and right eye were 0.48° (*SD* = 0.19°) and 0.47° (*SD* = 0.19°), respectively. Participants then went through the experimental trials. The presentation of original and horizontally mirrored sequences was blocked and block order was counterbalanced across participants. To enhance the number of observations per experimental condition, each block of original and mirrored sequences was presented twice consecutively (i.e., “2 × original–2 × mirrored” or “2 × mirrored–2 × original”). Within blocks, order of videos was newly randomized for each participant. However, the order in the second block of original or mirrored clips was the same random order as in the first block of respective type of clips to ensure that identical clips were the same distance apart (i.e., a video that was shown first in block 1 was also shown first in block 2, etc.). Overall, participants completed a total of 64 experimental trials.

Prior to each trial, a drift check was conducted by requiring participants to fixate on the center of a circle in the middle of the screen and to then press the spacebar. The maximum allowed error for fixation deviation from the position of the circle during drift check was set to the eye-tracking system's default value of 2° (left eye: *M* = 0.7°, *SD* = 0.39°; right eye: *M* = 0.7°, *SD* = 0.3°). Upon completion of the drift check the next video was shown and the monitor turned black 40 ms before the moment of ball release. The participants' task was identical to the familiarization trials. However, in the experimental trials neither feedback nor any other information that might have revealed a penalty's actual outcome was given. Collectively, testing took about 20 min for each participant.

### Measures

#### Prediction accuracy

For each participant, the percentage of correct predictions was calculated according to the correct corner (25% chance), side and height (50% chance each) separately for left- and right-handed penalties.

#### Response time

The time difference (in ms) between the end of a video and the moment of a participant's key press was considered as response time. Median response times were calculated for each participant separately for left- and right-handed penalties.

#### Gaze measures

The software Data Viewer (*SR Research*) was used to create fixation reports based on raw gaze data recorded from video onset until video offset in each trial for each participant. These reports enable the output of various parameters associated with a fixation such as a fixation's sequential number in a trial, its duration or the x-y-coordinates of a fixation, separately for both eyes, in screen pixel coordinates. Reports were based on the following settings specified in the Data Viewer. First, a threshold of 120 ms as minimum fixation duration was set. Second, nearby consecutive fixations were merged if a fixation's duration was below that threshold and if the distance between the two fixations was within 1°.

Following the creation of a fixation report, the data was further prepared for later analyses. First, for each trial's final fixation, recorded fixation offset was checked whether it was beyond the end of a video. This was necessary to ensure that the sum of individual fixation durations in a trial did not exceed the duration of a video shown in that trial. For almost any trial (99.88% of 2432 trials), the end time of such fixation was replaced with the value for video duration in the respective trial. Then, fixation duration was recalculated as the time difference between fixation onset and the new fixation offset and it was checked if that difference was larger than the fixation duration threshold of 120 ms. If not, that particular fixation was excluded from all further analyses (which happened in 15.63% of all trials), the fixation listed before was newly classified as the final fixation and the number of fixations for such trial was corrected accordingly (i.e., minus one). Second, the offset values recorded during drift check prior to each trial separately for the left and right eye were used to correct raw gaze data by subtracting offset values from raw data. On rare occasions (2.26% out of 2432 trials) these offset values indicated deviation from the screen's center larger than 2° and data of those trials were excluded from the analyses later on^1^. Third, for each fixation where data for both the left and right eye were available the binocular point of gaze was determined through calculation of the mean of the x- and y-coordinates available for both eyes. For time points where data were available for one eye only (8.39% out of 7,198,720 data points), the data for that eye was not considered as representative for binocular gaze coordinates because this might have introduced a possible bias (e.g., toward right or left). Finally, the duration of each binocular fixation was checked against the threshold of 120 ms for fixation duration. If that threshold was not met, the particular data was excluded from the following analyses (0.98% of 14,729 binocular fixations were excluded). Based on the gaze dataset resulting from the above steps, the *number of fixations* and *mean fixation duration* (in ms) were determined for each trial in each participant and calculated separately for left- vs. right-handed penalties.

Further, we explored whether there were differences in *final fixation duration* as a function of the participants' skill and/or the penalty-takers' handedness. To this end, we analyzed the duration of final fixations that lasted until the end of a video. Final fixations with an offset prior to the end of a video were excluded from analysis; resulting in the inclusion of 78.11% of all final fixations (goalkeepers: 76.36%; non-goalkeepers: 79.85%).

A graphical approach was used for the analysis and interpretation of the time-course of *horizontal fixation deviation* from the center of the screen^2^. To this end, first binocular gaze coordinates of shorter videos (i.e., duration of 2920 or 2960 ms) were aligned with the end of videos that lasted 3000 ms. This step was necessary to ensure that later averaging of fixation coordinates across different trials or videos was done for similar phases in the penalty-takers' movements. Second, the horizontal fixation deviation from the center of the screen was calculated through subtraction of 640 px from the x-coordinates of binocular fixations. Accordingly, negative (positive) values indicate fixations toward the left (right) half from the screen's center (e.g., see Nuthmann and Matthias, 2014, for a similar procedure). Then, for each participant the mean horizontal fixation deviation in the course of videos showing left- vs. right-handed penalties was calculated. Based on these data, the time-course of mean horizontal fixation deviations (i.e., from video onset to video offset, in ms) against left- and right-handed penalties and the corresponding 95% confidence intervals were finally determined separately for goalkeepers and non-goalkeepers. Since the content of videos showing left- and right-handed penalties was controlled through presentation of original and horizontally mirrored clips, symmetry of these time-courses along zero (i.e., the screen's midline) would indicate that participants adapted their gaze behavior to the penalty-takers' handedness.

### Data analysis

Given the aim and design of the experiment, analyses focused on the factors Skill (goalkeepers vs. non-goalkeepers; between-subject) and Throwers' Handedness (left vs. right; within-subject) and their impact on performance (i.e., prediction accuracy, response time) and gaze measures (i.e., number of fixations, fixation duration overall, final fixation duration and horizontal fixation deviation from the center of the screen) as defined above. To check for the factors' overall effects on prediction accuracy, response time, number of fixations, overall and final fixation duration, separate 2 (Skill) × 2 (Thrower's Handedness) ANOVAs with repeated measures on the last factor were run using SPSS (version 22). Alpha level was set at 5% and ANOVA effect sizes were calculated as partial eta-squared values ($\eta_{\text{p}}^{2}$).

## Results

Table 2 provides a summary of ANOVA results for prediction accuracy, response time, number of fixations, overall and final fixation duration.

**Table 2:** **Results from 2 × 2 mixed ANOVAs on prediction accuracy (corner, side, and height), response time, number of fixations, overall and final fixation duration**.

**Variable**	**Effect**	***F***	***p***	**$\eta_{p}^{2}$**	**1-β**
%-correct (corner)	Skill	35.224	< 0.001	0.495	1.00
	Hand	5.005	0.032	0.122	0.586
	Skill × Hand	1.599	0.214	0.043	0.234
%-correct (side)	Skill	43.679	< 0.001	0.548	1.00
	Hand	4.770	0.036	0.117	0.566
	Skill × Hand	1.192	0.282	0.032	0.186
%-correct (height)	Skill	8.944	0.005	0.199	0.829
	Hand	2.528	0.121	0.066	0.340
	Skill × Hand	0.596	0.445	0.016	0.117
Response time (ms)	Skill	8.619	0.006	0.193	0.815
	Hand	0.827	0.369	0.022	0.144
	Skill × Hand	0.001	0.973	< 0.001	0.050
Number of fixations	Skill	< 0.001	0.992	< 0.001	0.050
	Hand	0.438	0.513	0.012	0.099
	Skill × Hand	0.204	0.654	0.006	0.072
Fixation duration overall (ms)	Skill	0.009	0.925	< 0.001	0.051
	Hand	1.669	0.205	0.044	0.242
	Skill × Hand	0.820	0.371	0.022	0.143
Final fixation duration (ms)	Skill	2.263	0.141	0.059	0.310
	Hand	0.402	0.530	0.011	0.095
	Skill × Hand	1.686	0.202	0.045	0.244

*α = 5% and df = (1, 36) for all comparisons*.

### Prediction accuracy

Goalkeepers' and non-goalkeepers' accuracy for corner, side and height predictions against left- and right-handed penalty-takers are shown in Figures 1B,C. Overall, goalkeepers (GK) outperformed non-goalkeepers (Non-GK) in each direction prediction (corner: *M*_GK_ = 38.82%, *SD*_GK_ = 8.84% vs. *M*_Non−GK_ = 24.67%, *SD*_Non−GK_ = 5.45%; side: *M*_GK_ = 66.04%, *SD*_GK_ = 8.82% vs. *M*_Non−GK_ = 50.90%, *SD*_Non−GK_ = 4.67%; height: *M*_GK_ = 57.32%, *SD*_GK_ = 6.55% vs. *M*_Non−GK_ = 51.15%, *SD*_Non−GK_ = 6.16%). Further, left-handed shots were harder to predict than right-handed shots for corner (*M*_LH_ = 29.85%, *SD*_LH_ = 9.71% vs. *M*_RH_ = 33.63%, *SD*_RH_ = 12.99%) and side (*M*_LH_ = 56.33%, *SD*_LH_ = 11.52% vs. *M*_RH_ = 60.61%, *SD*_RH_ = 12.45%).

Figure 1B shows mean prediction accuracies against pairs of identical, as related to content, right- vs. left-handed penalties separately for corner, side and height predictions in goalkeepers (triangles) and non-goalkeepers (disks). The distribution of symbols relative to the blue diagonal line, which represents equal performance against left- and right-handed penalties, suggests that the handedness effect tended to be more consistent in goalkeepers than non-goalkeepers for corner and side predictions. Specifically, in the majority of pairs of penalties, goalkeepers achieved higher accuracy against a right- than left-handed version of an action, as evidenced by most symbols being located clearly below the diagonal. In non-goalkeepers, the distribution of symbols representing video pairs was more balanced in relation to the diagonal. This difference in goalkeepers and non-goalkeepers is also reflected in larger standardized effect sizes associated with accuracy differences against left- vs. right-handed actions in goalkeepers as opposed to novices (see values noted above bars in Figure 1C). Conversely, for height predictions the effect tended to be larger in non-goalkeepers than goalkeepers (Figure 1C). None of the two-way interactions in ANOVAs, however, indicated a statistical difference for any of the three direction predictions (Table 2).

Visual inspection of 95% confidence intervals associated with mean accuracy scores in Figure 1C indicates that goalkeepers, but not non-goalkeepers, performed above chance level for corner, side and height predictions against both left- and right-handed penalties.

### Response time

ANOVA revealed that, on average, goalkeepers (*M* = 991.47 ms, *SD* = 374.27 ms) responded earlier than non-goalkeepers (*M* = 1521.45 ms, *SD* = 692.19 ms). Furthermore, response times did not considerably differ against left-handed (*M* = 1274.22 ms, *SD* = 666.27 ms) and right-handed penalties (*M* = 1238.69 ms, *SD* = 575.33 ms) and this pattern was almost the same in goalkeepers and non-goalkeepers (Figure 1D).

### Number of fixations, overall and final fixation duration

**Figure 2:**
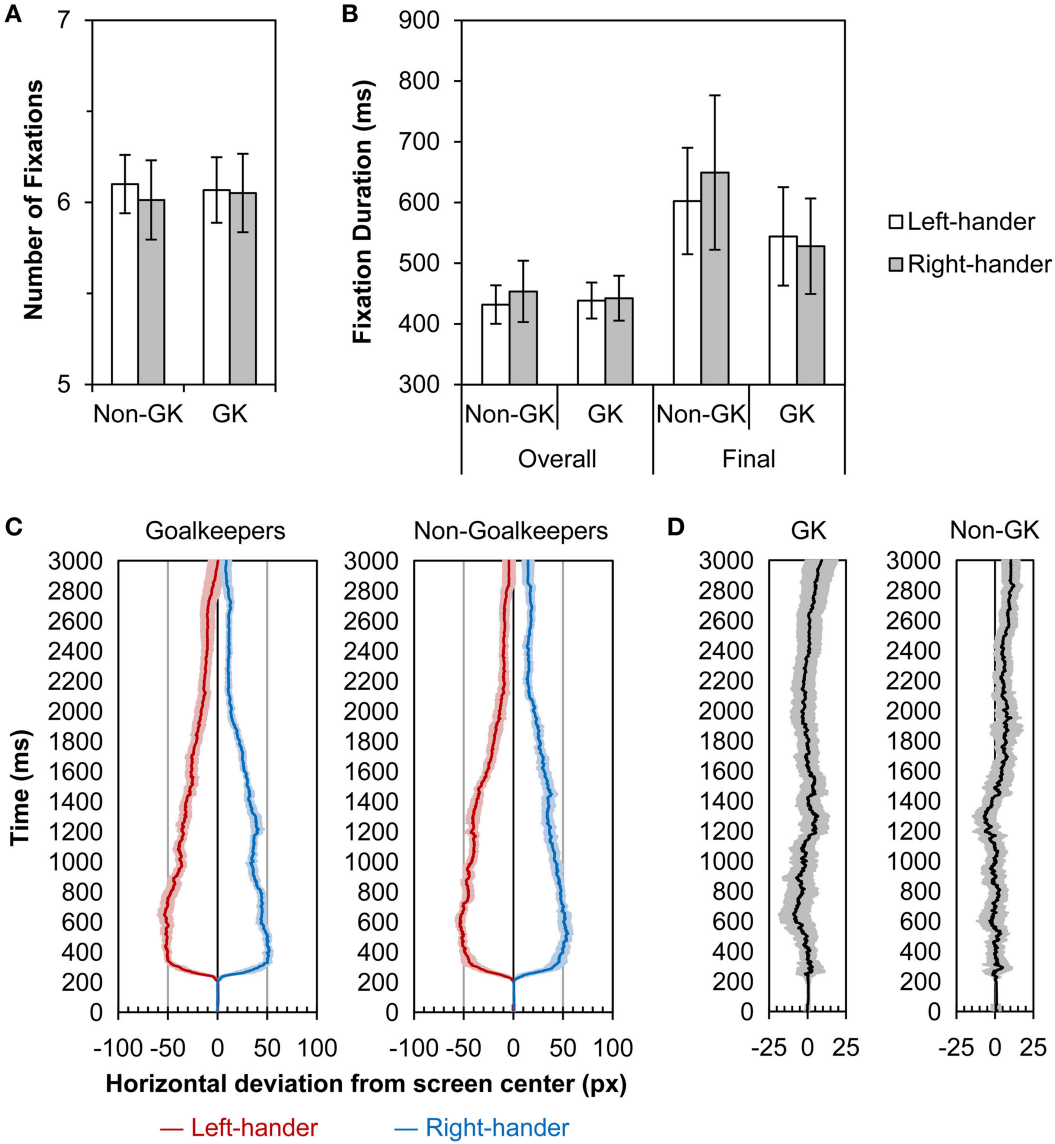
**(A)** Mean number of fixations and **(B)** mean fixation duration overall and for final fixation against left- and right-handed penalties separately for goalkeepers and non-goalkeepers. In panels **(A,B)** error bars represent 95% confidence intervals associated with each mean. **(C)** Time-course of mean horizontal fixation deviation from the center of the screen against left- (red) vs. right-handed (blue) penalties separately for goalkeepers and non-goalkeepers. Red and blue shaded areas represent 95% confidence intervals associated with respective means. **(D)** Time-course of the difference in mean horizontal fixation deviation from the center of the screen between right- and left-handed penalties separately for goalkeepers (GK) and non-goalkeepers (Non-GK). Gray shaded areas represent 95% confidence intervals associated with mean differences.

Goalkeepers' and non-goalkeepers' mean number of fixations as well as mean overall and final fixation duration against left- and right-handed penalties are illustrated in Figures 2A,B. For each variable, ANOVA did not reveal statistical differences as a function of the factors Skill or Throwers' Handedness neither in isolation nor in combination (see Table 2).

### Time-course of horizontal fixation deviation

Figure 2C illustrates the time-course of horizontal fixation deviation from the center of the screen when left- and right-handed penalty-takers were viewed, separately for goalkeepers and non-goalkeepers. Overall and apart from subtle variations, the time-courses for left- and right-handed penalties were quite symmetric along the screen's midline. Fixations close to zero at the beginning of time-courses (i.e., approximately first 200 ms) originate from a drift check which required participants to visually fixate on a centrally presented circle prior to the start of a video (see Section Procedure for details).

To illustrate further, horizontal fixation deviations for left- and right-handed actions were overlaid and the mean differences in these deviations were calculated (Figure 2D). To this end, the time-course of deviation values for left-handed penalties was mirrored through multiplication with “−1,” such that differences were obtained via the formula *x*_RH_ − (−1)^*^x_LH_. Consequently, positive differences indicate that the mean horizontal fixation position against right-handed throws was, from an observer's perspective, to the right relative to the mean horizontal fixation position against left-handed throws. Conversely, negative differences indicate that the mean horizontal fixation position against right-handed throws was, from an observer's perspective, to the left relative to the mean horizontal fixation position against left-handed throws. Zero, in turn, indicates no difference in mean horizontal fixation positions against right- and left-handed penalties. As is shown in Figure 2D, mean differences oscillate closely along zero in both goalkeepers and non-goalkeepers, indicating almost no distinct differences in horizontal fixation orientation between left- and right-handed penalties.

**Figure 3:**
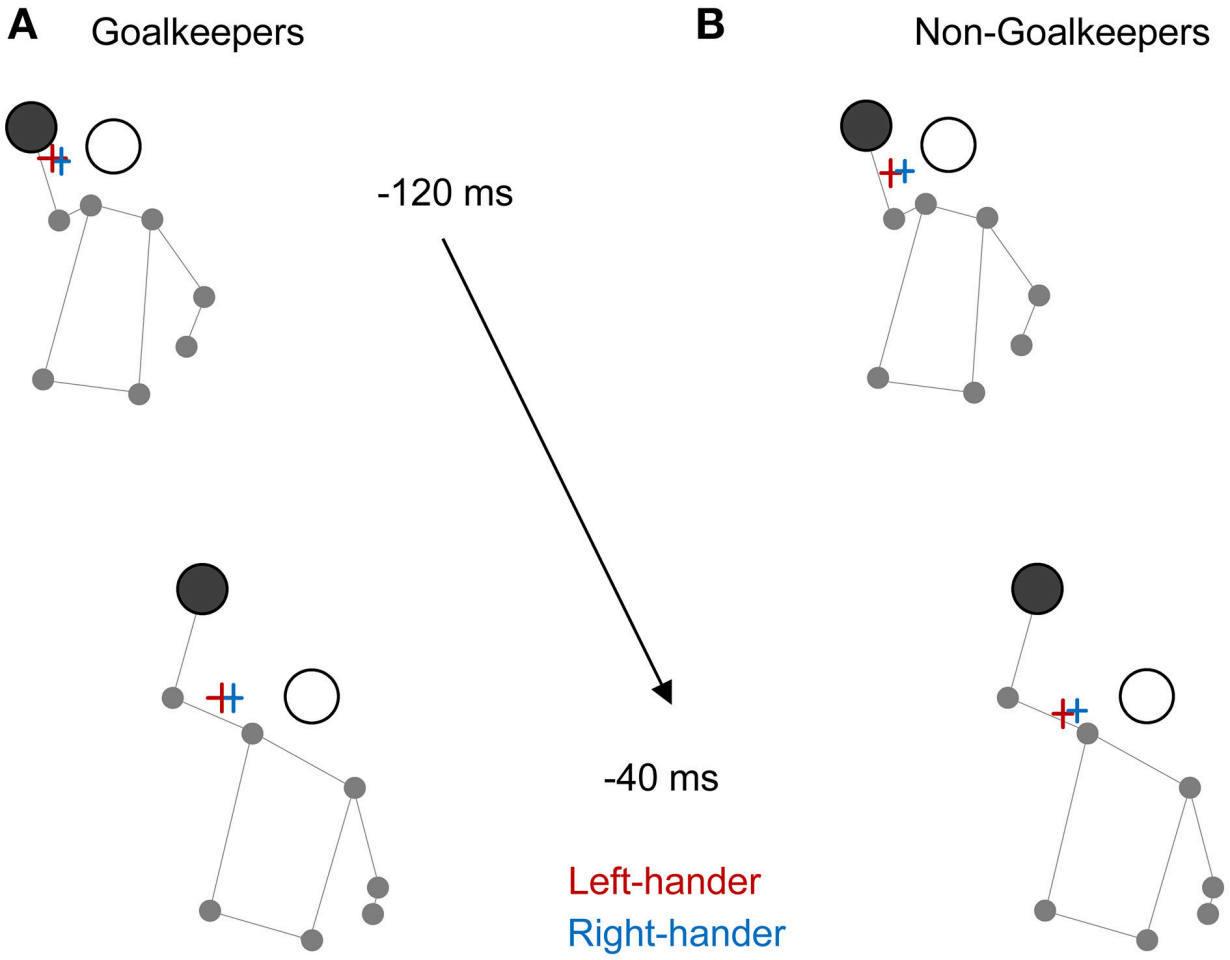
**Illustration of mean fixation positions relative to a penalty-taker's body at two different time points (−120 and −40 ms) close to the end of a video in (A) goalkeepers and (B) non-goalkeepers**. While right-handed penalty-takers are illustrated, please note that mean fixation positions are displayed for both right- (blue) and left-handed (red) shots. For illustrative purposes, fixation values for left-handed shots were superimposed on right-handed shots by multiplication of originally recorded values with “−1.” The lengths of horizontal and vertical bars correspond to 95% confidence intervals associated with mean horizontal and vertical fixation positions (as represented by an intersection of error bars), respectively.

In non-goalkeepers, there was a trend for slight differences (up to about 10 px) toward the end of videos from around 1800 ms onwards (see the course of means and associated 95% confidence intervals in Figure 2D). To further explore this issue, in Figures 3A,B goalkeepers' and non-goalkeepers' mean fixation positions against left- (red) and right-handed (blue) penalties are exemplarily displayed for the antepenultimate (−120 ms) and the finale frame (−40 ms) of videos. For illustrative purposes and to facilitate comparison of fixation locations, fixation values for left-handed shots were superimposed on right-handed shots by multiplication of originally recorded values with “−1.” In non-goalkeepers, there could be some slight handedness-dependent differences in horizontal gaze orientation, with mean fixation location being further away from a left- than right-handed penalty-takers' body (see Figure 3B). However, it is important to consider that 10 px corresponded to ~0.30°, that the eye-tracking system's average accuracy is 0.25–0.5° according to the manufacturer's instructions and that mean absolute average errors measured during the eye-tracker's validation in our experiment were 0.48° for both eyes. Thus, the small differences illustrated in Figures 2D, 3A,B were within the margin of error of gaze measurements. Therefore, we will refrain from elaborating on this issue in the following discussion.

## Discussion

Previous research demonstrated athletes' superior anticipation of right-handed as opposed to left-handed action outcomes (Hagemann, 2009; Loffing et al., 2012b, 2015). In general, low familiarity with relatively rarer left-handed individuals has been suggested as a key mechanism underlying this effect (Schorer et al., 2012). However, whether particular perceptual-cognitive processes such as gaze behavior differ during observation and anticipation of left- vs. right-handed actions is far from being understood. To this end, in a video-based experiment we examined goalkeepers' and non-goalkeepers' accuracy and gaze behavior in visual anticipation of left- vs. right-handed penalties in team-handball.

### Left- vs. right-handed penalties

In line with our hypothesis and consistent with previous findings, left-handed action outcomes were more difficult to anticipate than right-handed action outcomes. Such handedness effect in accuracy, however, was not paralleled by distinct handedness-dependent differences in response time or gaze behavior. With regard to the latter, neither discrete (i.e., number of fixations, overall or final fixation duration) nor dynamic gaze measures (i.e., time-course of horizontal fixation deviations from the center of the screen) varied considerably as a function of a penalty-taker's handedness (see the Supplementary Material online for time-courses of mean vertical fixation deviation).

The absence of misalignment in participants' gaze when viewing left-handed actions (e.g., directing gaze preferentially toward an opponent's right body side; Neumaier, 1983) seems to conflict with the idea that perceptual asymmetries, which are possibly caused and/or reinforced by the predominance of human right-handedness, may explain lower anticipation accuracy against left-handed athletes (Marzoli et al., 2014, 2015). Instead, the time-courses of horizontal fixation deviation indicate that participants, on average, adjusted their gaze almost symmetrical along the midline for left- and right-handed actions (Figures 2C,D). Furthermore, there were also no differences in the mean number of fixations or overall and final fixation durations against left- and right-handed penalties. Though speculative, one might have assumed that observers' low familiarity with left-handed actions could have resulted in a more scattered visual search pattern against left-handed penalties—i.e., higher number of fixations of shorter duration—as compared to right-handed penalties. Such prediction could come from findings on expert-novice differences in visual search behavior, suggesting that novices (who are perceptually less familiar with domain-specific actions than experts) employ, on average, a less economic gaze strategy of more fixations and shorter duration (Mann et al., 2007; Gegenfurtner et al., 2011). This issue could be addressed in future work using a refined experimental design (see also Section Study Limitations, Alternative Explanations, and Research Perspectives below).

So far, our findings suggest that observers may not necessarily employ different visual search strategies when confronted with left- or right-handed opponents, but they may be differently capable of picking up and interpreting the visual information available for predicting future states of left- vs. right-handed actions. In this regard, analogous to explanations for performance differences between experts and novices in domain-specific perceptual tasks (e.g., Yarrow et al., 2009), observers' low perceptual familiarity with left-handed individuals may limit access to representations or templates of left-handed actions and thereby hinder categorization of such actions with accuracy comparable to more familiar right-handed actions (Clotfelter, 2008; Hagemann, 2009).

### Goalkeepers vs. non-goalkeepers

With regard to expertise differences, our findings conform to research demonstrating superior visual anticipation of action intentions in skilled compared to less skilled or novice participants (for reviews e.g., see Williams, 2009; Müller and Abernethy, 2012). Goalkeepers clearly outperformed non-goalkeepers in corner, side and height predictions. Also, on average goalkeepers responded considerably earlier than non-goalkeepers (see also Savelsbergh et al., 2002). However, our data did not reveal skill differences in gaze measures (Mann et al., 2007) such as in number of fixations, fixation duration overall and final fixation duration. Likewise, the time-courses of mean horizontal (Figure 2C) and vertical fixation deviation (Figure S1 in the Supplementary Material online) as well as fixation locations toward the end of videos (Figures 3A,B) against both left- and right-handed penalty-takers were very similar in both skill groups. These data suggest that, while goalkeepers and non-goalkeepers directed their gaze to similar locations, they were differently capable of using the visual information for inferring a penalty's outcome. We will address the question of why gaze measures did not differ between skill groups in the following section.

### Study limitations, alternative explanations, and research perspectives

Some limitations as well as alternative explanations may apply to the issues discussed above. First, to some extent the absence of distinct handedness-dependent differences in gaze behavior could be specific to the actions presented in our experiment. For penalty-throw movements, the trajectories of a penalty-taker's body parts and the ball are highly predictable. Also, observers may have little difficulty computing the location from where the ball will leave a player's hand and orient their gaze accordingly, irrespective of whether the right or left hand is used for throwing (see Figures 3A,B). Thus, we speculate that such “ball-in-the-hand”-effect may render difficult the detection of distinct handedness-dependent differences in gaze behavior in team-handball penalties. In contrast, in sports like volleyball or tennis, where the interplay between a player's movement and the approaching ball has to be observed carefully and where the position of hand- or racket-ball-contact and resulting ball flight need to be inferred from their relative motions, distinct differences in gaze orientation against left- vs. right-handed opponents may be more likely to occur and possibly explain accuracy differences in visual anticipation (Hagemann, 2009; Loffing et al., 2012b, 2015). The aforementioned scenario could also be one explanation for why goalkeepers' and non-goalkeepers' gaze behavior did not differ considerably.

Second, use of a static testing environment where visual perception was decoupled from normally required interceptive action in goalkeeping may also have prevented the detection of handedness and/or skill differences in gaze behavior. Surely, this is a relevant concern in light of the theory of two visual streams (Milner and Goodale, 2008) and the concerns raised about findings from perception-action-decoupled experimental research on visual anticipation in sports (e.g., van der Kamp et al., 2008; Mann et al., 2010). Consequently, we acknowledge that replication of our experiment in more representative settings seems warranted. Penalties could be presented as life-size projections in the laboratory (Savelsbergh et al., 2002; Mann et al., 2013) or testing could take place *in-situ* on the field; in both cases using mobile eye-tracking devices and asking participants to move in the direction they anticipate a penalty to go (e.g., Dicks et al., 2010).

Third, the presentation of penalties on a 19″ computer monitor may have restricted the occurrence of variation in participants' gaze. In the experiment, the height of penalty-takers shown in the videos corresponded to 12.5–14° of visual angle (depending on the individual penalty-takers' size). This is close to the visual angle when goalkeepers stand 7 m away on the goal-line while awaiting a penalty of players who are between 1.8 and 2 m in height (angle: 14.42–15.95°). However, since in reality goalkeepers are allowed to position themselves between the goal-line and a penalty-taker up to a distance of 4 m away from the goal-line, and often apply this strategy to increase the goal area covered by their body, a penalty-taker's height then covers larger visual angle on a goalkeeper's retina than we were able to realize with the equipment used in the experiment. Hence, the absence of differences in gaze behavior depending on participants' skill or penalty-takers' handedness might be due to the limited size of videos shown. On the other hand, at least for team-handball goalkeeping, inclusion of mobile devices and more realistic life-size projections as well as requiring participants to move must not ultimately result in skill differences in gaze measures (Schorer, 2007).

Fourth, we did not control or manipulate the amount of participants' familiarity with left- vs. right-handed actions. Instead, we based our hypotheses on the assumption that participants would be considerably less familiar with left-handed actions because of the predominance of right-handedness in the normal or handball population (Gilbert and Wysocki, 1992; Loffing et al., 2014). To determine the impact of varying perceptual familiarity with left- or right-handed movements on gaze or other process measures in more detail, future experiments should employ a pre-post design with interim perceptual training where participants are confronted either with left- or right-handed actions only (cf. Schorer et al., 2012).

Finally, even if the above limitations were perfectly solved it could still turn out that gaze strategies do not considerably differ against left- and right-handed opponents. Therefore, another approach could be to examine the potential differential contribution of left- vs. right-handed opponents' body regions (e.g., arms, shoulder, hips) to visual anticipation of their action intentions, for example, through the presentation of spatially manipulated penalties (Bourne et al., 2013; Loffing and Hagemann, 2014). Along with the specification of the regions from where athletes are likely to have most difficulties picking up anticipation-relevant information in left-handed actions, this could help to better understand left-right asymmetries in the prediction of action intentions in human social interaction as well as to develop appropriate perceptual training intervention.

## Author contributions

Conceived and designed the experiment: FL, FS, NH, BS. Performed the experiment: FL, FS. Analyzed the data: FL. Wrote the paper: FL, FS, NH, BS.

## Funding

This work was supported by a research grant awarded by the German Research Foundation (DFG) to NH (HA 4361/5-2) and BS (STR 490/11-2). The funders had no role in study design, data collection and analysis, decision to publish, or preparation of the manuscript.

### Conflict of interest statement

The authors declare that the research was conducted in the absence of any commercial or financial relationships that could be construed as a potential conflict of interest.

## Footnotes

^1^Please note that inclusion of gaze data from all trials irrespective of drift offset values does not lead to results considerably different to those reported here.

^2^Since penalty-takers' handedness in the video should particularly affect participants' horizontal fixation deviation, we focus on that measure in the main text. We additionally analyzed vertical fixation deviation from the center of the screen and readers interested in these analyses are referred to the Supplementary Material online.

## References

[B1] Azémar G. Ripoll H. Simonet P. Stein J. F. (1983). Étude neuro-psychologique du comportement des gauchers en escrime. Cinésiologie 22, 7–18.

[B2] Bideau B. Multon F. Kulpa R. Fradet L. Arnaldi B. Delamarche P. (2004). Using virtual reality to analyze links between handball thrower kinematics and goalkeeper's reactions. Neurosci. Lett. 372, 119–122. 10.1016/j.neulet.2004.09.023 15531100

[B3] Bourne M. Bennett S. J. Hayes S. J. Smeeton N. J. Williams A. M. (2013). Information underpinning anticipation of goal-directed throwing. Attent. Percept. Psychophys. 75, 1559–1569. 10.3758/s13414-013-0485-2 23801321

[B4] Brooks R. Bussière L. F. Jennions M. D. Hunt J. (2004). Sinister strategies succeed at the cricket World Cup. Proc. Biol. Sci. 271, S64–S66. 10.1098/rsbl.2003.0100 15101421 PMC1809987

[B5] Cañal-Bruland R. Schmidt M. (2009). Response bias in judging deceptive movements. Acta Psychol. 130, 235–240. 10.1016/j.actpsy.2008.12.009 19193359

[B6] Chokron S. De Agostini M. (2000). Reading habits influence aesthetic preference. Brain Res. Cogn. Brain Res. 10, 45–49. 10.1016/s0926-6410(00)00021-5 10978691

[B7] Clotfelter E. D. (2008). Frequency-dependent performance and handedness in professional baseball players. J. Comp. Psychol. 122, 68–72. 10.1037/0735-7036.122.1.68 18298283

[B8] Dicks M. Button C. Davids K. (2010). Examination of gaze behaviors under *in situ* and video simulation task constraints reveals differences in information pickup for perception and action. Attent. Percept. Psychophys. 72, 706–720. 10.3758/app.72.3.706 20348577

[B9] Edwards S. Beaton A. (1996). Howzat? Why is there an over-representation of left-handed bowlers in professional cricket in the UK? Laterality 1, 45–50. 15513028 10.1080/713754208

[B10] Fagard J. Dahmen R. (2004). Cultural influences on the development of lateral preferences: a comparison between French and Tunisian children. Laterality 9, 67–78. 10.1080/13576500342000167 15382731

[B11] Faurie C. Schiefenhövel W. Le Bomin S. Billiard S. Raymond M. (2005). Variation in the frequency of left-handedness in traditional societies. Curr. Anthropol. 46, 142–147. 10.1086/427101

[B12] Friedrich T. E. Harms V. L. Elias L. J. (2014). Dynamic stimuli: accentuating aesthetic preference biases. Laterality 19, 549–559. 10.1080/1357650x.2014.886585 24527986

[B13] Gegenfurtner A. Lehtinen E. Saljo R. (2011). Expertise differences in the comprehension of visualizations: a meta-analysis of eye-tracking research in professional domains. Educ. Psychol. Rev. 23, 523–552. 10.1007/s10648-011-9174-7

[B14] Gilbert A. N. Wysocki C. J. (1992). Hand preference and age in the United States. Neuropsychologia 30, 601–608. 10.1016/0028-3932(92)90065-T 1528408

[B15] Goldstein S. R. Young C. A. (1996). “Evolutionary” stable strategy of handedness in major league baseball. J. Comp. Psychol. 110, 164–169. 10.1037/0735-7036.110.2.164

[B16] Grouios G. (2004). Motoric dominance and sporting excellence: training versus heredity. Percept. Mot. Skills 98, 53–66. 10.2466/pms.98.1.53-66 15058866

[B17] Hagemann N. (2009). The advantage of being left-handed in interactive sports. Attent. Percept. Psychophys. 71, 1641–1648. 10.3758/App.71.7.1641 19801623

[B18] Jewell G. McCourt M. E. (2000). Pseudoneglect: a review and meta-analysis of performance factors in line bisection tasks. Neuropsychologia 38, 93–110. 10.1016/s0028-3932(99)00045-7 10617294

[B19] Llaurens V. Raymond M. Faurie C. (2009). Why are some people left-handed? An evolutionary perspective. Philos. Trans. R. Soc. Lond. B Biol. Sci. 364, 881–894. 10.1098/rstb.2008.0235 19064347 PMC2666081

[B20] Lobinger B. Büsch D. Werner K. Pabst J. Gail S. Sichelschmidt P. (2014). Erfolgsrelevante Aktionsmuster von Torhütern beim Siebenmeterwurf im Spitzenhandball [Analysis of action patterns of goalkeepers in 7-meter-throw-ins in top-level handball]. Z. Sportpsychol. 21, 74–85. 10.1026/1612-5010/a000116

[B21] Loffing F. Hagemann N. (2012). Side bias in human performance: a review on the left-handers' advantage in sports, in Bias in Human Behaviour, eds Dutta T. Mandal M. Kumar S. (Hauppauge, NY: Nova Science), 163–182.

[B22] Loffing F. Hagemann N. (2014). Skill differences in visual anticipation of type of throw in team-handball penalties. Psychol. Sport Exerc. 15, 260–267. 10.1016/j.psychsport.2014.01.006

[B23] Loffing F. Hagemann N. Schorer J. Baker J. (2015). Skilled players' and novices' difficulty anticipating left- vs. right-handed opponents' action intentions varies across different points in time. Hum. Mov. Sci. 40, 410–421. 10.1016/j.humov.2015.01.018 25689236

[B24] Loffing F. Hagemann N. Strauss B. (2012a). Left-handedness in professional and amateur tennis. PLoS ONE 7:e49325. 10.1371/journal.pone.0049325 23145151 PMC3492260

[B25] Loffing F. Schorer J. Hagemann N. Baker J. (2012b). On the advantage of being left-handed in volleyball: further evidence of the specificity of skilled visual perception. Attent. Percept. Psychophys. 74, 446–453. 10.3758/s13414-011-0252-1 22147534

[B26] Loffing F. Sölter F. Hagemann N. (2014). Left preference for sport tasks does not necessarily indicate left-handedness: sport-specific lateral preferences, relationship with handedness and implications for laterality research in behavioural sciences. PLoS ONE 9:e105800 10.1371/journal.pone.0105800 25141020 PMC4139391

[B27] Maass A. Pagani D. Berta E. (2007). How beautiful is the goal and how violent is the fistfight? Spatial bias in the interpretation of human behavior. Soc. Cogn. 25, 833–852. 10.1521/soco.2007.25.6.833

[B28] Mann D. L. Abernethy B. Farrow D. (2010). Action specificity increases anticipatory performance and the expert advantage in natural interceptive tasks. Acta Psychol. 135, 17–23. 10.1016/j.actpsy.2010.04.006 20507831

[B29] Mann D. L. Spratford W. Abernethy B. (2013). The head tracks and gaze predicts: how the world's best batters hit a ball. PLoS ONE 8:e58289. 10.1371/journal.pone.0058289 23516460 PMC3596397

[B30] Mann D. T. Y. Williams A. M. Ward P. Janelle C. M. (2007). Perceptual-cognitive expertise in sport: a meta-analysis. J. Sport Exerc. Psychol. 29, 457–478. 17968048 10.1123/jsep.29.4.457

[B31] Marzoli D. Lucafò C. Pagliara A. Cappuccio R. Brancucci A. Tommasi L. (2015). Both right- and left-handers show a bias to attend others' right arm. Exp. Brain Res. 233, 415–424. 10.1007/s00221-014-4124-5 25318614

[B32] Marzoli D. Prete G. Tommasi L. (2014). Perceptual asymmetries and handedness: a neglected link? Front. Psychol. 5:163. 10.3389/fpsyg.2014.00163 24592250 PMC3938099

[B33] McManus I. C. (2002). Right Hand, Left Hand: The Origins of Asymmetry in Brains, Bodies, Atoms and Culture. London: Weidenfeld & Nicolson.

[B34] McMorris T. Colenso S. (1996). Anticipation of professional soccer goalkeepers when facing right- and left-footed penalty kicks. Percept. Mot. Skills 82, 931–934. 10.2466/pms.1996.82.3.931

[B35] Milner A. D. Goodale M. A. (2008). Two visual systems re-viewed. Neuropsychologia 46, 774–785. 10.1016/j.neuropsychologia.2007.10.005 18037456

[B36] Müller S. Abernethy B. (2012). Expert anticipatory skill in striking sports: a review and a model. Res. Q. Exerc. Sport 83, 175–187. 10.5641/027013612800745059 22808703

[B37] Neumaier A. (1983). Beobachtungsstrategien und Antizipation bei der Abwehr von Volleyballangriffen [Observational strategies and anticipation during the defense of volleyball attacks]. Leistungssport 13, 5–10.

[B38] Nicholls M. E. R. Loftus A. Mayer K. Mattingley J. B. (2007). Things that go bump in the right: the effect of unimanual activity on rightward collisions. Neuropsychologia 45, 1122–1126. 10.1016/j.neuropsychologia.2006.07.015 16999981

[B39] Nuthmann A. Matthias E. (2014). Time course of pseudoneglect in scene viewing. Cortex 52, 113–119. 10.1016/j.cortex.2013.11.007 24388005

[B40] Oldfield R. C. (1971). The assessment and analysis of handedness: the Edinburgh inventory. Neuropsychologia 9, 97–113. 5146491 10.1016/0028-3932(71)90067-4

[B41] Perelle I. B. Ehrman L. (1994). An international study of human handedness: the data. Behav. Genet. 24, 217–227. 10.1007/BF01067189 7945152

[B42] Raymond M. Pontier D. Dufour A. B. Møller A. P. (1996). Frequency-dependent maintenance of left handedness in humans. Proc. Biol. Sci. 263, 1627–1633. 10.1098/rspb.1996.0238 9025310

[B43] Savelsbergh G. J. P. Williams A. M. Van der Kamp J. Ward P. (2002). Visual search, anticipation and expertise in soccer goalkeepers. J. Sports Sci. 20, 279–287. 10.1080/026404102317284826 11999482

[B44] Schorer J. (2007). Höchstleistung im Handballtor - Eine Studie zur Identifikation, den Mechanismen und der Entwicklung senso-motorischer Expertise [High performance in handball goals - A study on the identification, mechanisms, and development of sensory-motor expertise]. Dissertation, Ruprecht-Karls-Universität Heidelberg. Retrieved from: http://www.ub.uni-heidelberg.de/archiv/7310

[B45] Schorer J. Baker J. (2009). An exploratory study of aging and perceptual-motor expertise in handball goalkeepers. Exp. Aging Res. 35, 1–19. 10.1080/03610730802544641 19173099

[B46] Schorer J. Cobley S. Büsch D. Bräutigam H. Baker J. (2009). Influences of competition level, gender, player nationality, career stage and playing position on relative age effects. Scand. J. Med. Sci. Sports 19, 720–730. 10.1111/j.1600-0838.2008.00838.x 18627551

[B47] Schorer J. Loffing F. Hagemann N. Baker J. (2012). Human handedness in interactive situations: negative perceptual frequency effects can be reversed! J. Sports Sci. 30, 507–513. 10.1080/02640414.2012.654811 22296164

[B48] van der Kamp J. Rivas F. van Doorn H. Savelsbergh G. (2008). Ventral and dorsal system contributions to visual anticipation in fast ball sports. Int. J. Sport Psychol. 39, 100–130.

[B49] Voyer D. Voyer S. D. Tramonte L. (2012). Free-viewing laterality tasks: a multilevel meta-analysis. Neuropsychology 26, 551–567. 10.1037/a0028631 22731609

[B50] Werner S. L. Guido J. A. Delude N. A. Stewart G. W. Greenfield J. H. Meister K. (2010). Throwing arm dominance in collegiate baseball pitching: a biomechanical study. Am. J. Sports Med. 38, 1606–1610. 10.1177/0363546510365511 20543146

[B51] Williams A. M. (2009). Perceiving the intentions of others: how do skilled performers make anticipation judgments?, in Progress in Brain Research, eds Raab M. Johnson J. G. Heekeren H. R. (Amsterdam: Elsevier), 73–83. 10.1016/S0079-6123(09)01307-7 19477331

[B52] Yarrow K. Brown P. Krakauer J. W. (2009). Inside the brain of an elite athlete: the neural processes that support high achievement in sports. Nat. Rev. Neurosci. 10, 585–596. 10.1038/nrn2672 19571792

