# Supplementary material for: Accuracy of Outcome Anticipation, But Not Gaze Behavior, Differs Against Left- and Right-Handed Penalties in Team-Handball Goalkeeping
Source: Front Psychol. 2015 Dec 1;6:1820. doi: 10.3389/fpsyg.2015.01820 (PMC4664728; doi:10.3389/fpsyg.2015.01820)
Supplement: Supplementary file 1 [file Image1.PDF]

Vertical fixation deviation from the center of the screen

For sake of completeness, in addition to the time-courses of mean horizontal fixation deviation reported in the main text (see Fig. 2C) we calculated the time-courses of mean vertical fixation deviation from the screen's center. The data is illustrated in Figure S1 for left- and right-handed penalties separately for goalkeepers and non-goalkeepers.
As for horizontal fixation deviation, the data do not indicate any considerable difference in gaze orientation between left- and right-handed penalties in any of the two skill groups.

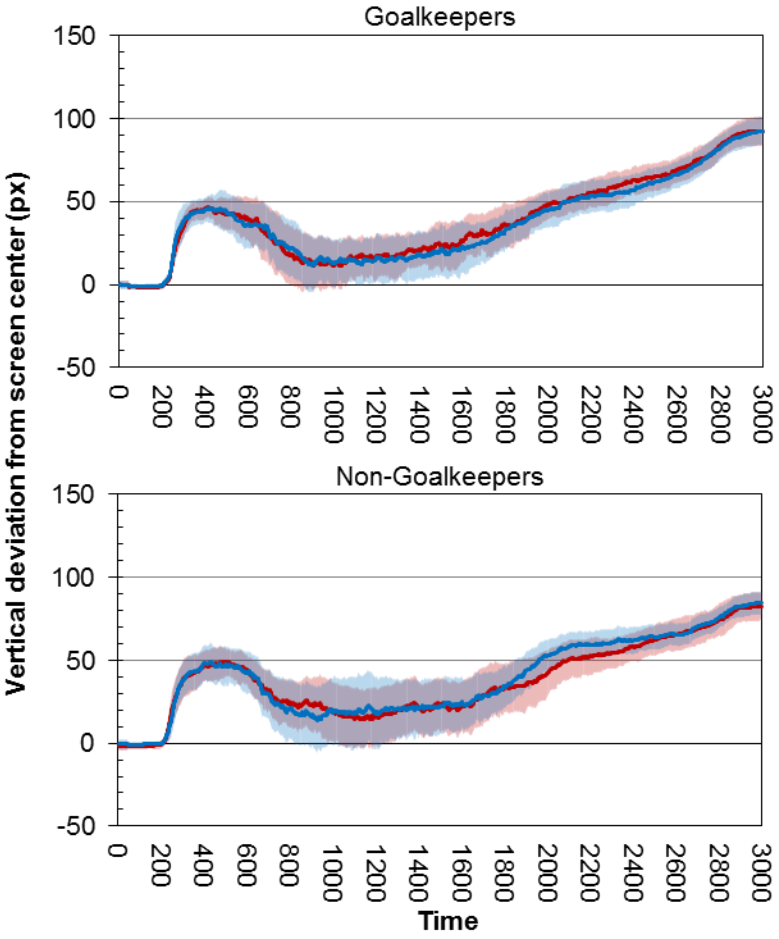

Figure S1. Time-course of mean vertical fixation deviation from the center of the screen against left- (red) vs. right-handed (blue) penalties separately for goalkeepers (top) and non-goalkeepers (bottom). Red and blue shaded areas represent 95% confidence intervals associated with respective means. Fixations close to zero at the beginning of time-courses (i.e., approximately first 200 ms) originate from a drift check which required participants to visually fixate on a centrally presented circle. Positive (negative) values on the ordinate indicate fixations in the upper (lower) half of the screen.
